# Combatting Antibiotic Resistance: Identifying Gaps in Knowledge, Attitude, and Practice Among Medical Interns

**DOI:** 10.7759/cureus.64402

**Published:** 2024-07-12

**Authors:** Jiyana Bano, Ishita Gupta, Gaurav Singh, Sheikh M Abdur Rahman, R. Narsimha Rao, Ekta Yadav, Brajendra Singh, Karnika Agrawal, Surendra Kumar

**Affiliations:** 1 Pediatrics, Maharaja Agrasen Medical College, Hisar, IND; 2 General Medicine, Maharaja Agrasen Medical College, Hisar, IND; 3 Neonatology, Himalayan Institute of Medical Sciences, Dehradun, IND

**Keywords:** medical curriculum, practice, attitude, knowledge, interns, antibiotic stewardship, antibiotic prescription, antibiotics, antibiotic resistance

## Abstract

Introduction: Antibiotic resistance presents a significant global health threat to modern medicine. The awareness and attitude of future doctors undergoing training may play a crucial role in addressing this important issue, influencing the control of resistance and promoting responsible antibiotic stewardship. This study aimed to estimate knowledge, attitudes, and practices regarding antibiotic usage and antimicrobial resistance among tertiary care teaching hospital medical interns.

Methodology: The questionnaire-based cross-sectional study was conducted on 123 MBBS interns from multiple medical institutions. Intern’s knowledge, attitudes, and self-reported practices regarding antibiotic use were recorded.

Results: Based on survey responses from 123 participants, 116 (94.31%) were aware of the adverse effects of indiscriminate antibiotic use, recognizing the risks of ineffective treatment, increased adverse effects, prolonged illness, bacterial resistance, and higher medical costs. Most (106, 86.18%) acknowledged the challenges of treating antibiotic-resistant infections, and 69 (56.10%) correctly identified that bacteria are not a cause of the common cold and flu. Most (115, 93.5%) recognized antibiotic resistance as a significant global health problem. In attitude, 90 (73%) believed antibiotics should be avoided for colds, but 80 (65%) thought they hastened fever recovery. Only 48 (39%) recognized that antibiotics contribute to resistance, while 102 (83%) agreed skipping doses fosters resistance. Most support hospital policies (118, 96%) and curriculum courses (112, 91%) for rational antibiotic use. Regarding practice, 12 (9.76%) interns admitted to overusing antibiotics, 68 (55.28%) consulted a doctor before starting antibiotics, and 87 (70.73%) checked expiry dates. Additionally, 62 (50.41%) preferred antibiotics for cough and sore throat symptoms.

Conclusions: The study highlights that while interns have a good knowledge and awareness of the harms of antibiotic misuse, they are not translating this knowledge into practice. This indicates a disconnect between understanding and application. Therefore, there is a need to add a rational antibiotic prescription and stewardship module to the medical curriculum to ensure that knowledge is effectively translated into changing beliefs and practices.

## Introduction

The global escalation of antimicrobial resistance, including within India, poses a significant threat to public health. Rapid emergence and dissemination of new resistance mechanisms challenge our capacity to effectively combat common infectious diseases. Mitigating the incidence and advancement of antibiotic resistance is paramount, representing an urgent imperative [1].

A primary catalyst for drug resistance is the widespread misuse of antibiotics, a pervasive issue worldwide. This misuse fosters the proliferation of multi-drug-resistant organisms (MDROs), contributing to prolonged hospitalizations, heightened morbidity, and increased mortality rates associated with MDRO-induced infections. Heightened awareness of the gravity and implications of antimicrobial resistance serves as a foundational step in its management [2].

Prescribers assume a pivotal role in the battle against antimicrobial resistance, not solely through judicious prescription practices but also by fostering patient awareness regarding safe antibiotic utilization. Their proactive engagement is instrumental in curbing the proliferation of drug-resistant pathogens and safeguarding public health [1-5].

The inadequacy of training provided during medical education may contribute to a lack of confidence in undertaking essential tasks. Therefore, integrating comprehensive training on the proper prescription and usage of antibiotics into the MBBS curriculum is crucial, given the frequent utilization of antibiotics in clinical practice. However, assessing the target population's baseline knowledge, attitudes, and practices is essential before any modifications or additional educational initiatives can be taken.

Understanding these factors will facilitate the development of a tailored and effective curriculum. Few studies have been conducted in the past on undergraduates of various disciplines including MBBS, pharmacy, nursing and physiotherapy students and interns to assess their awareness and perceptions on antibiotic use, out of which some have shown a gap in their knowledge and practice [6-17]. In this context, our study aims to evaluate the knowledge, attitudes, and practices regarding antibiotic usage and resistance among MBBS interns at various medical colleges in Haryana. MBBS interns were chosen as participants because they have completed their MBBS curriculum training and are preparing for their future roles as healthcare practitioners in society. It is crucial for them to have a strong baseline knowledge and follow rational antibiotic prescription practices to serve the community better and help prevent the global issue of widespread antibiotic resistance. This study was planned and aimed to estimate the knowledge, attitude, and practices of MBBS interns regarding antibiotic use and antimicrobial resistance in tertiary care teaching hospitals. The study will provide valuable insights to inform curriculum enhancements and educational interventions aimed at addressing antibiotic resistance effectively.

## Materials and methods

This was a cross-sectional descriptive study conducted during a period from December 2023 to March 2024 over MBBS interns of medical colleges of Haryana. It examined the knowledge, attitudes, and practices of antibiotic use among MBBS interns. A structured questionnaire was distributed to the interns, and those who completed and submitted the questionnaire were included in the study. The questionnaire was prepared with the help of previous studies and validated by a pilot study on 15 participants. The exclusion criteria included all interns who declined to give consent for participation in the study. Therefore, a convenience sample of MBBS interns who filled out the questionnaire from various medical institutions was recruited for participation. A minimum sample size of 59 was obtained based on 90% prevalence in a study by Akalu and Belavadi, with a 95% confidence level and precision of 5%, by online, web-based software, OpenEpi, version 3, the open-source calculator developed by CDC [6,18]. However, we included all 123 interns who filled out and submitted the questionnaire to further increase the power of the study. Data collection was facilitated through structured questionnaires created online using Google Forms, which were subsequently disseminated to participants via text messages, WhatsApp messages, and emails. Informed written consent was incorporated in the Google Form. The questionnaire aimed at eliciting information on several key domains: intern's demographic characteristics, their knowledge regarding antibiotic use, their attitude toward preventive antibiotic use, and self-reported practices concerning antibiotic prescription. The questionnaire was structured into four distinct sections: demography, knowledge, attitude, and practice. In the demographic section age and sex of the intern were documented. In the knowledge section, respondents were required to answer four closed-ended questions using the options true, false, or maybe. The attitude section featured seven statements with response options ranging from strongly agree to strongly disagree, allowing interns to express their opinions on preventive antibiotic use. Finally, the practice section required interns to indicate their practices using response options of yes, no, or sometimes by four closed-ended questions. The questionnaire used in this study is provided in the Appendix. The collected data were stored in a password-protected file. The categorical variables were summarized as percentages. Data were analyzed by descriptive statistics by online, web-based software, OpenEpi, version 3, the open-source calculator developed by CDC [18]. The study was carried out after obtaining approval from the Scientific Research Committee and the Institutional Ethics Committee for Human Research.

## Results

**Figure 1:**
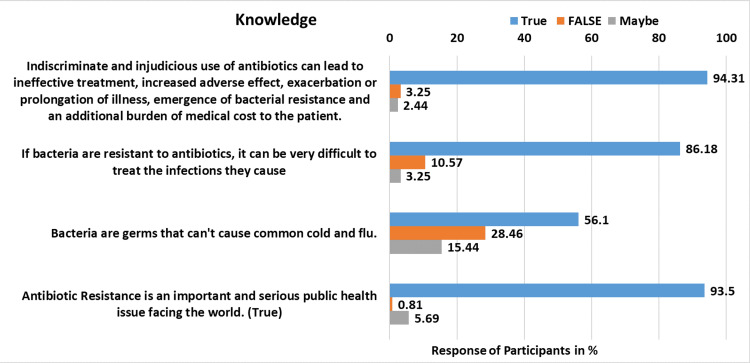
Response of participants for knowledge questions.

A total of 123 responses were received, comprising 52 females and 71 males. Responses for four knowledge-related questions are detailed in Table 1 and illustrated in Figure 1. A vast majority, comprising 116 (94.31%) respondents, exhibited a strong awareness of the adverse consequences associated with indiscriminate and injudicious antibiotic use. They recognized that such practices can lead to ineffective treatment, heightened adverse effects, exacerbation or prolongation of illness, emergence of bacterial resistance, and increased medical costs for patients. In contrast, a small minority of 4 (3.25%) expressed skepticism regarding these outcomes, while 3 (2.44%) remained uncertain. Moreover, when presented with the challenge of treating infections caused by antibiotic-resistant bacteria, most candidates (106, 86.18%) correctly acknowledged the difficulty associated with such cases. However, a noteworthy portion (13, 10.57%) of respondents erroneously believed this statement to be false, while 4 (3.25%) expressed uncertainty. Opinions varied regarding the misconception that bacteria are responsible for causing the common cold and flu. While 69 (56.10%) respondents correctly identified this statement as false, a significant proportion (35, 28.46%) erroneously claimed it to be true and 19 (15.44%) remained uncertain. Finally, when asked about the significance of antibiotic resistance as a global public health issue, the majority (115, 93.5%) recognized its gravity. However, a marginal fraction (1, 0.81%) disagreed with this assertion and 7 (5.69%) expressed uncertainty.

**Table 1:** Medical intern’s knowledge about antibiotic uses.

Questions (Correct answer)	True (%)	False (%)	Maybe (%)
Indiscriminate and injudicious use of antibiotics can lead to ineffective treatment, increased adverse effects, exacerbation or prolongation of illness, the emergence of bacterial resistance, and the additional burden of medical cost to the patient. (True)	116 (94.31)	04 (3.25)	03 (2.44)
If bacteria are resistant to antibiotics, it can be very difficult to treat the infections they cause. (True)	106 (86.18)	13 (10.57)	04 (3.25)
Bacteria are germs that cannot cause the common cold and flu. (True)	69 (56.10)	35 (28.46)	19 (15.44)
Antibiotic resistance is an important and serious public health issue facing the world. (True)	115 (93.5)	01 (0.81)	07 (5.69)

**Figure 2:**
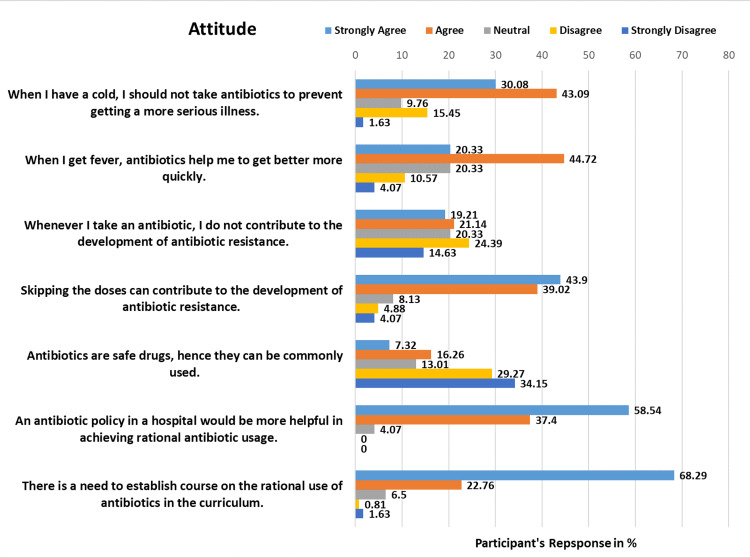
Response of participants regarding attitude statments

The analysis of Table 2 reveals insights into the attitude section, where participants were queried on various aspects of antibiotic usage. Among the 123 respondents, the distribution of responses is shown in Figure 2. Reactions varied when asked whether antibiotics should be avoided for colds to prevent more serious illnesses. Specifically, 37 (30.08%) strongly agreed, 53 (43.09%) agreed, 12 (9.76%) were neutral, 19 (15.45%) disagreed, and 2 (1.62%) strongly disagreed. Regarding the belief that antibiotics hasten recovery from fever, 25 (20.32%) strongly agreed, 55 (44.71%) agreed, 25 (20.33%) were neutral, 13 (10.57%) disagreed, and 5 (4.07%) strongly disagreed. The perception that antibiotic usage does not contribute to resistance yielded varied responses. Specifically, 24 (19.21%) strongly agreed, 26 (21.14%) agreed, 25 (20.33%) were neutral, 30 (24.39%) disagreed, and 18 (14.63%) strongly disagreed. To the question of whether skipping antibiotic doses can foster resistance, 54 (43.90%) strongly agreed, 48 (39.02%) agreed, 10 (8.13%) were neutral, 6 (4.88%) disagreed, and 5 (4.07%) strongly disagreed. Regarding the perception of antibiotics as universally safe drugs, 9 (7.32%) strongly agreed, 20 (16.26%) agreed, 16 (13.01%) were neutral, while 36 (29.27%) disagreed and 42 (34.15%) strongly disagreed. Participants largely supported the idea of implementing antibiotic policies in hospitals to promote rational usage. Specifically, 72 (58.54%) strongly agreed, 46 (37.40%) agreed, 5 (4.07%) were neutral, and no respondents disagreed or strongly disagreed. Lastly, the need for a curriculum course on the rational use of antibiotics received strong endorsement with 84 (68.29%) strongly agreeing, 28 (22.76%) agreeing, 8 (6.50%) neutral, 1 (0.81%) disagreeing, and 2 (1.62%) strongly disagreeing.

**Table 2:** Medical intern’s attitude toward antibiotic uses. SA, strongly agree; A, agree; N, neutral; D, disagree; SD, strongly disagree

Attitude statements	SA (%)	A (%)	N (%)	D (%)	SD (%)
When I have a cold, I should not take antibiotics to prevent getting a more serious illness.	37 (30.08)	53 (43.09)	12 (9.76)	19 (15.45)	02 (1.62)
When I get fever, antibiotics help me to get better more quickly.	25 (20.32)	55 (44.71)	25 (20.33)	13 (10.57)	05 (04.07)
Whenever I take an antibiotic, I do not contribute to the development of antibiotic resistance.	24 (19.21)	26 (21.14)	25 (20.33)	30 (24.39)	18 (14.63)
Skipping the doses can contribute to the development of antibiotic resistance.	54 (43.90)	48 (39.02)	10 (08.13)	06 (04.88)	05 (04.07)
Antibiotics are safe drugs, hence they can be commonly used.	09 (07.32)	20 (16.26)	16 (13.01)	36 (29.27)	42 (34.15)
An antibiotic policy in a hospital would be more helpful in achieving rational antibiotic usage.	72 (58.54)	46 (37.40)	05 (04.07)	00 (00)	00 (00)
There is a need to establish a course on the rational use of antibiotics in the curriculum.	84 (68.29)	28 (22.76)	08 (06.50)	01 (0.81)	02 (1.62)

**Figure 3:**
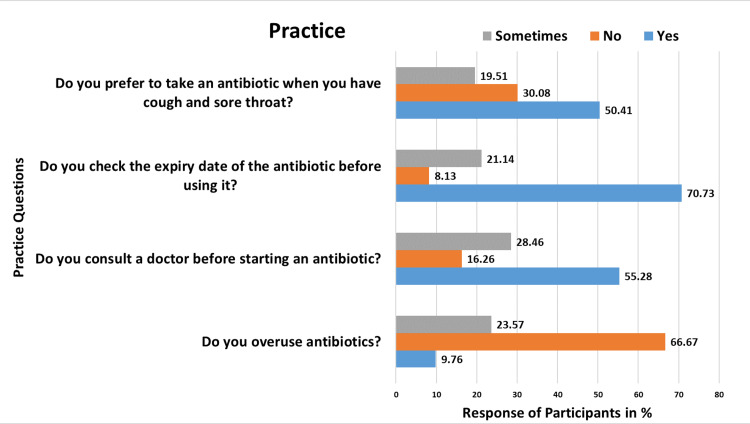
Responses from participants for practice questions.

Table 3 provides insights into the practices concerning antibiotic usage, as respondents were queried on various aspects and responses presented in Figure 3. When asked whether they overuse antibiotics, 12 (9.76%) admitted to doing so, 82 (66.67%) indicated they do not, and 29 (23.58%) responded with sometimes. Regarding consulting a doctor before starting an antibiotic, 68 (55.28%) answered that they seek medical advice, 20 (16.26%) stated that they do not consult a doctor, and 35 (28.46%) responded with sometimes, indicating occasional reliance on professional guidance. When questioned about checking the expiry date of antibiotics before usage, 87 (70.73%) confirmed that they do so, while 10 (8.13%) admitted to not checking, and 26 (21.14%) responded with sometimes. Lastly, respondents were asked about their preference for taking antibiotics when experiencing symptoms like cough and sore throat. Of the respondents, 62 (50.40%) acknowledged a tendency to opt for antibiotics, 37 (30.08%) stated they did not, and 24 (19.51%) responded with sometimes.

**Table 3:** Medical intern's practice regarding antibiotic use.

Questions	Yes (%)	No (%)	Sometimes (%)
Do you overuse antibiotics?	12 (09.76)	82 (66.67)	29 (23.58)
Do you consult a doctor before starting an antibiotic?	68 (55.28)	20 (16.26)	35 (28.46)
Do you check the expiry date of the antibiotic before using it?	87 (70.73)	10 (08.13)	26 (21.14)
Do you prefer to take an antibiotic when you have cough and sore throat?	62 (50.41)	37 (30.08)	24 (19.51)

## Discussion

A meta-analysis by Murray et al. provided a comprehensive assessment of the global burden of anti-microbial resistance and concluded that it is a leading cause of death worldwide, with the highest burden in low-resource settings [5]. In an attempt to find out the current understanding of antibiotic resistance in the future doctors of society, our study highlighted that 94.31% of MBBS interns exhibited a strong awareness of the adverse consequences associated with indiscriminate and injudicious antibiotic use and 93.5% recognized the significance of antibiotic resistance as a global public health problem, which is consistent with the findings of Kulkarni et al. and Akalu and Belavadi [6,7]. The majority (96%) of the interns in the study of Kulkarni et al. believed that antibiotic resistance is a serious global health issue [7]. In the study by Akalu and Belavadi on 75 interns, there was a unanimous belief (100%) among interns that indiscriminate antibiotic use leads to antimicrobial resistance [6]. In our study, only 56.10% of the respondents knew that bacteria are not responsible for causing the common cold and flu, which is in contrast to the study by Akalu and Belavadi in which 90.6% of the interns were aware of the fact that these are due to viral etiology and not bacterial [6]. However, in the study of Khan et al. on medical students, a notable 22.7% of participants lacked awareness regarding the viral etiology of colds and flu, mistakenly attributing them to bacterial causes [8].

However, when it comes to attitude, only 73% agreed that antibiotics should be avoided for colds whereas in Akalu and Belavadi’s study, 69 (92%) thought that antibiotics are not the first choice for cough and sore throat [6]. In the study of Ganesh M et al, which assessed and compared the knowledge, attitude, and practices concerning antibiotic prescription and resistance of medical interns, final MBBS students, and physiotherapy students, it was found that about 55% of medical interns believed that antibiotics can cure viral infections and 72% believed that antibiotics speed up the recovery of cough and cold, which reflect even a poorer score than ours [9]. Also, in the study by Huang et al from China which recruited about 1250 clinical medical students and 1250 paired non-medical respondents, 64.5% of medical students believed that antibiotics could cure viral infections and 27.4% thought that the use of antibiotics would speed up the recovery of cold and cough [10]. Lv et al from China found that more than 10% of undergraduate students from various disciplines incorrectly believed that antibiotics should be used for common colds [11]. In a study by Sakeena et al conducted in 2018 over 466 pharmacy students, more than half (57%) of junior pharmacy students incorrectly believed antibiotics were suitable for cold and flu management [12]. In the study conducted by Khan et al in 2013, findings revealed that approximately 38% of medical students advocated for the use of antibiotics upon developing a cold, with 60% believing it would expedite their recovery [8]. The common inference from all these studies is clearly that medical students and interns are not aware of the fact that common colds and flu are caused by viruses and antibiotics should not be used in their management. Unnecessary use will aggravate the problem of antimicrobial resistance.

In our study, 66% disagreed that antibiotics are universally safe drugs, similar to the findings of Akalu and Belavadi where 66.6% disagreed [6]. Additionally, Khan et al found that 15.5% of participants regarded antibiotics as safe for common use, implying a potential misunderstanding of their appropriate usage [8]. Alarmingly 40% in our study believed that antibiotic use does not contribute to resistance which is against the findings of Ganesh et al. and Huang et al. where 97% and 87.9%, respectively, knew that frequent use of antibiotics decreased their efficacy; 93% and 90.1% of participants, respectively, believed that there is an overuse of antibiotics at present and it is leading to bacterial resistance [9,10]. In Lv et al.'s study, 79.1% of students believed that limiting the inappropriate use of antibiotics played an important role in preventing the emergence of antibiotic resistance [11].

Despite this, one positive aspect is that in our study, a large majority supported implementing antibiotic policies in hospitals to promote their rational use (96%) and expressed the need for a curriculum course on rational antibiotic usage (91%). Similarly, in the study by Kulkarni et al., 82% strongly felt the need for a hospital antibiotic policy [7]. In the study by Ganesh et al also, 90% of medical interns appreciated the need to establish a course on the rational use of antibiotics at the university [9]. Huang et al. also found that 89.2% wanted to get more education about antibiotics and 74.2% recognized the need for such a course [10]. We found that nearly 82% of intern doctors understood that skipping antibiotic doses should be avoided as it can foster resistance similar to the findings of Akalu and Belavadi and Kulkarni et al. where 62.6% and 60%, respectively, recognized the risk of antibiotic resistance with missed doses [6,7]. Therefore, despite having an adequate knowledge base, the attitude that reflects their beliefs and influences their behavior or practice needs to be changed to achieve rational antibiotic prescription and effective stewardship practices.

Taking into consideration their self-reported practices regarding antibiotic use, approximately 33% of the participants admitted to overusing antibiotics. Around 45% reported not always seeking professional guidance before starting an antibiotic, whereas in the study of Ganesh et al., 77% of interns consulted the doctors for the prescription of antibiotics [9]. This signifies that many times antibiotics are self-prescribed, which is deleterious. Additionally, a large number of our medical interns (70%) indicated a tendency to opt for antibiotics instead of seeking professional evaluation when experiencing symptoms of cough and sore throat, which is in contrast to Akalu and Belavadi in which 69 (92%) thought that antibiotics are the not the first choice for cough and sore throat [6]. About 25% of interns in the study of Ganesh et al. and 13.6% of medical students in the study of Huang et al. used antibiotics when they experienced the common cold, which again contributed to antibiotic misuse [9,10].

These results highlight significant gaps between knowledge and practice, emphasizing the need for improved education and adherence to guidelines to ensure responsible antibiotic use, prevent misuse, and effectively combat antibiotic resistance. This gap between knowledge, attitude, and practice is prevalent among students of all fields, including nursing, pharmacy, dental, and medical programs, and is observed globally [6-17]. If these future healthcare providers misuse antibiotics on themselves, it is unlikely they will avoid misusing them with their patients, as their practice reflects their strong beliefs. To bring a change in these beliefs or attitudes, the curriculum needs to be further strengthened, enabling them to fully understand the importance of this issue.

Knowledge alone is not sufficient to change behavior, but it plays a crucial role in shaping beliefs and attitudes regarding that behavior. There is a need for targeted educational interventions for interns to promote responsible antibiotic prescribing practices, mitigate antibiotic resistance, and safeguard public health. Incorporating rational antibiotic prescription and antibiotic stewardship into the MBBS curriculum can contribute to the goal of reducing antibiotic resistance. Huang et al. and Asharani et al. also recommended a change in the curriculum of MBBS to fill this gap between knowledge and practice of antibiotic prescription and stewardship [10,17].

The strength of our study was the conduction of the study on intern doctors from various colleges who have completed their undergraduate training so that it can assess the effect of the whole undergraduate medical curriculum on their knowledge, perception, and practices. Many MBBS interns are often the first contact physicians for patients at primary health care centers or busy OPDs, so it is essential to improve their practices regarding antibiotic use to promote antibiotic stewardship and prevent the emergence of MDROs that do not respond to existing antibiotics. The study's limitations include its descriptive design, self-reported data, and the use of a convenient sampling method. These issues could be addressed by conducting a pre-post-intervention study or an interventional study with randomization to generate higher quality evidence in the future.

## Conclusions

After evaluating the responses of interns regarding antibiotic use, we concluded that although there was awareness among interns regarding the adverse consequences of indiscriminate antibiotic use and challenges posed by antibiotic-resistant bacteria in treatment, a notable proportion exhibited misconceptions regarding the role of bacteria in causing common colds and flu. The study also revealed insights into interns' attitudes toward antibiotic usage, highlighting varied beliefs and perceptions. While a significant number supported the implementation of antibiotic policies in hospitals and the incorporation of courses on rational antibiotic use in the curriculum, diverse opinions were noted regarding the safety and necessity of antibiotics for upper respiratory symptoms. Furthermore, an analysis of respondents' practices underscored areas for improvement, such as the need for consistent consultation with doctors before antibiotic use and vigilance in checking expiry dates. Notably, a significant percentage admitted to occasional overuse of antibiotics.

These findings emphasize the importance of ongoing education and awareness initiatives to foster responsible antibiotic prescribing practices among medical professionals. Addressing misconceptions, promoting evidence-based decision-making, and encouraging judicious antibiotic use by including rational antibiotic prescription and antibiotic stewardship in the undergraduate medical curriculum are imperative in mitigating antibiotic resistance and safeguarding public health. Continued research and interventions in this domain are warranted to address the multifaceted challenges posed by antibiotic resistance.

## Appendices

Appendix

**Table 4:** Master chart for questions and participants' responses.

S. No.	Age?	Sex?	Q.1 Indiscriminate and injudicious use of antibiotics can lead to: ineffective treatment, increased adverse effect, exacerbation or prolongation of illness, the emergence of bacterial resistance and the additional burden of medical cost to the patient. (True)	Q.2 If bacteria are resistant to antibiotics, it can be very difficult to treat the infections they cause (True)	Q.3 Bacteria are germs that can't cause common cold and flu. (True)	Q.4 Antibiotic resistance is an important and serious public health issue facing the world. (True)	Q.5 When I have a cold, I should not take antibiotics to prevent getting a more serious illness.	Q.6 When I get fever, antibiotics help me to get better more quickly.	Q.7 Whenever I take an antibiotic, I do not contribute to the development of antibiotic resistance.	Q.8 Skipping the doses can contribute to the development of antibiotic resistance.	Q.9 Antibiotics are safe drugs; hence they can be commonly used.	Q. 10 An antibiotic policy in a hospital would be more helpful in achieving rational antibiotic Usage.	Q. 11 There is a need to establish course on the ‘rational use of antibiotics’ in the curriculum.	Q.12 Do you overuse antibiotics?	Q.13 Do you consult a doctor before starting an antibiotic?	Q.14 Do you check the expiry date of the antibiotic before using it?	Q.15 Do you prefer to take an antibiotic when you have cough and sore throat?
1	25 - 34	Male	FALSE	FALSE	May be	TRUE	A. Strongly agree	A. Strongly agree	A. Strongly agree	A. Strongly agree	B. Agree	B. Agree	A. Strongly agree	A. Yes	A. Yes	C. Sometimes	A. Yes
2	18 - 24	Female	TRUE	TRUE	TRUE	TRUE	D. Disagree	B. Agree	C. Neutral	A. Strongly agree	D. Disagree	B. Agree	B. Agree	B. No	C. Sometimes	A. Yes	C. Sometimes
3	18 - 24	Male	TRUE	TRUE	TRUE	TRUE	C. Neutral	A. Strongly agree	A. Strongly agree	A. Strongly agree	B. Agree	A. Strongly agree	A. Strongly agree	B. No	B. No	A. Yes	A. Yes
4	18 - 24	Female	TRUE	TRUE	TRUE	TRUE	B. Agree	B. Agree	A. Strongly agree	A. Strongly agree	B. Agree	A. Strongly agree	A. Strongly agree	B. No	A. Yes	A. Yes	A. Yes
5	18 - 24	Female	TRUE	TRUE	TRUE	TRUE	A. Strongly agree	C. Neutral	E. Strongly disagree	A. Strongly agree	E. Strongly disagree	A. Strongly agree	A. Strongly agree	B. No	A. Yes	A. Yes	C. Sometimes
6	18 - 24	Female	TRUE	TRUE	TRUE	TRUE	B. Agree	B. Agree	C. Neutral	A. Strongly agree	E. Strongly disagree	A. Strongly agree	A. Strongly agree	B. No	A. Yes	A. Yes	C. Sometimes
7	18 - 24	Male	TRUE	TRUE	TRUE	TRUE	D. Disagree	C. Neutral	D. Disagree	B. Agree	E. Strongly disagree	A. Strongly agree	A. Strongly agree	B. No	C. Sometimes	A. Yes	C. Sometimes
8	18 - 24	Male	TRUE	TRUE	FALSE	TRUE	B. Agree	B. Agree	C. Neutral	A. Strongly agree	E. Strongly disagree	A. Strongly agree	E. Strongly disagree	B. No	A. Yes	A. Yes	B. No
9	25 - 34	Male	TRUE	FALSE	FALSE	TRUE	A. Strongly agree	B. Agree	C. Neutral	A. Strongly agree	B. Agree	A. Strongly agree	A. Strongly agree	B. No	A. Yes	B. No	A. Yes
10	25 - 34	Female	TRUE	FALSE	May be	TRUE	A. Strongly agree	B. Agree	C. Neutral	A. Strongly agree	B. Agree	C. Neutral	B. Agree	A. Yes	B. No	A. Yes	A. Yes
11	25 - 34	Male	TRUE	TRUE	TRUE	TRUE	A. Strongly agree	B. Agree	A. Strongly agree	B. Agree	A. Strongly agree	B. Agree	A. Strongly agree	B. No	A. Yes	A. Yes	A. Yes
12	18 - 24	Female	TRUE	TRUE	FALSE	TRUE	A. Strongly agree	A. Strongly agree	D. Disagree	B. Agree	E. Strongly disagree	B. Agree	A. Strongly agree	C. Sometimes	B. No	A. Yes	A. Yes
13	18 - 24	Male	TRUE	FALSE	TRUE	TRUE	B. Agree	A. Strongly agree	D. Disagree	B. Agree	C. Neutral	B. Agree	B. Agree	A. Yes	B. No	A. Yes	A. Yes
14	18 - 24	Male	TRUE	FALSE	May be	TRUE	A. Strongly agree	B. Agree	C. Neutral	A. Strongly agree	A. Strongly agree	A. Strongly agree	B. Agree	C. Sometimes	A. Yes	A. Yes	A. Yes
15	18 - 24	Male	TRUE	FALSE	FALSE	TRUE	A. Strongly agree	B. Agree	B. Agree	B. Agree	E. Strongly disagree	A. Strongly agree	A. Strongly agree	C. Sometimes	C. Sometimes	C. Sometimes	A. Yes
16	18 - 24	Male	TRUE	FALSE	TRUE	TRUE	B. Agree	A. Strongly agree	C. Neutral	C. Neutral	A. Strongly agree	A. Strongly agree	A. Strongly agree	A. Yes	C. Sometimes	B. No	A. Yes
17	25 - 34	Female	TRUE	TRUE	May be	TRUE	A. Strongly agree	A. Strongly agree	D. Disagree	B. Agree	B. Agree	A. Strongly agree	A. Strongly agree	C. Sometimes	B. No	A. Yes	A. Yes
18	25 - 34	Female	TRUE	TRUE	May be	TRUE	B. Agree	D. Disagree	A. Strongly agree	A. Strongly agree	B. Agree	A. Strongly agree	A. Strongly agree	C. Sometimes	A. Yes	C. Sometimes	A. Yes
19	18 - 24	Female	TRUE	TRUE	TRUE	FALSE	B. Agree	A. Strongly agree	C. Neutral	B. Agree	B. Agree	A. Strongly agree	A. Strongly agree	A. Yes	A. Yes	A. Yes	A. Yes
20	18 - 24	Male	FALSE	FALSE	TRUE	TRUE	D. Disagree	B. Agree	B. Agree	A. Strongly agree	A. Strongly agree	B. Agree	C. Neutral	A. Yes	B. No	B. No	C. Sometimes
21	18 - 24	Female	FALSE	TRUE	TRUE	TRUE	D. Disagree	C. Neutral	D. Disagree	D. Disagree	D. Disagree	B. Agree	A. Strongly agree	B. No	C. Sometimes	C. Sometimes	A. Yes
22	25 - 34	Female	TRUE	May be	TRUE	TRUE	A. Strongly agree	B. Agree	D. Disagree	B. Agree	D. Disagree	B. Agree	A. Strongly agree	C. Sometimes	C. Sometimes	A. Yes	A. Yes
23	18 - 24	Female	TRUE	TRUE	FALSE	TRUE	B. Agree	A. Strongly agree	E. Strongly disagree	A. Strongly agree	B. Agree	B. Agree	B. Agree	B. No	A. Yes	A. Yes	A. Yes
24	18 - 24	Female	FALSE	TRUE	FALSE	TRUE	B. Agree	A. Strongly agree	A. Strongly agree	E. Strongly disagree	D. Disagree	A. Strongly agree	A. Strongly agree	C. Sometimes	A. Yes	A. Yes	A. Yes
25	18 - 24	Male	TRUE	TRUE	May be	TRUE	D. Disagree	B. Agree	B. Agree	B. Agree	D. Disagree	B. Agree	A. Strongly agree	B. No	C. Sometimes	A. Yes	A. Yes
26	25 - 34	Male	TRUE	TRUE	FALSE	TRUE	B. Agree	D. Disagree	C. Neutral	B. Agree	E. Strongly disagree	B. Agree	B. Agree	B. No	C. Sometimes	C. Sometimes	C. Sometimes
27	18 - 24	Female	TRUE	TRUE	FALSE	TRUE	D. Disagree	B. Agree	E. Strongly disagree	B. Agree	B. Agree	B. Agree	A. Strongly agree	C. Sometimes	B. No	A. Yes	A. Yes
28	25 - 34	Male	TRUE	TRUE	TRUE	TRUE	D. Disagree	D. Disagree	B. Agree	B. Agree	B. Agree	B. Agree	A. Strongly agree	A. Yes	C. Sometimes	A. Yes	A. Yes
29	18 - 24	Female	TRUE	FALSE	TRUE	TRUE	B. Agree	A. Strongly agree	E. Strongly disagree	B. Agree	E. Strongly disagree	B. Agree	A. Strongly agree	B. No	C. Sometimes	A. Yes	C. Sometimes
30	18 - 24	Female	TRUE	TRUE	TRUE	TRUE	A. Strongly agree	B. Agree	C. Neutral	A. Strongly agree	E. Strongly disagree	A. Strongly agree	A. Strongly agree	B. No	A. Yes	A. Yes	C. Sometimes
31	18 - 24	Female	TRUE	TRUE	TRUE	TRUE	A. Strongly agree	B. Agree	D. Disagree	B. Agree	E. Strongly disagree	A. Strongly agree	A. Strongly agree	B. No	C. Sometimes	A. Yes	A. Yes
32	18 - 24	Female	TRUE	TRUE	May be	TRUE	D. Disagree	B. Agree	A. Strongly agree	E. Strongly disagree	E. Strongly disagree	A. Strongly agree	A. Strongly agree	B. No	C. Sometimes	A. Yes	B. No
33	18 - 24	Male	TRUE	TRUE	FALSE	TRUE	A. Strongly agree	B. Agree	B. Agree	B. Agree	E. Strongly disagree	B. Agree	B. Agree	C. Sometimes	B. No	A. Yes	B. No
34	18 - 24	Male	TRUE	TRUE	TRUE	TRUE	B. Agree	B. Agree	E. Strongly disagree	B. Agree	C. Neutral	B. Agree	A. Strongly agree	A. Yes	C. Sometimes	A. Yes	B. No
35	18 - 24	Male	TRUE	TRUE	TRUE	TRUE	A. Strongly agree	A. Strongly agree	A. Strongly agree	B. Agree	B. Agree	B. Agree	A. Strongly agree	B. No	B. No	A. Yes	A. Yes
36	25 - 34	Female	TRUE	TRUE	FALSE	TRUE	A. Strongly agree	B. Agree	E. Strongly disagree	B. Agree	E. Strongly disagree	A. Strongly agree	A. Strongly agree	C. Sometimes	B. No	A. Yes	A. Yes
37	18 - 24	Female	TRUE	TRUE	FALSE	TRUE	B. Agree	A. Strongly agree	A. Strongly agree	B. Agree	D. Disagree	A. Strongly agree	A. Strongly agree	B. No	B. No	C. Sometimes	A. Yes
38	18 - 24	Male	TRUE	TRUE	TRUE	TRUE	A. Strongly agree	B. Agree	A. Strongly agree	A. Strongly agree	C. Neutral	B. Agree	A. Strongly agree	B. No	A. Yes	A. Yes	B. No
39	18 - 24	Female	TRUE	TRUE	TRUE	TRUE	A. Strongly agree	B. Agree	C. Neutral	B. Agree	D. Disagree	A. Strongly agree	A. Strongly agree	B. No	C. Sometimes	A. Yes	C. Sometimes
40	18 - 24	Male	TRUE	TRUE	May be	TRUE	B. Agree	D. Disagree	B. Agree	A. Strongly agree	E. Strongly disagree	B. Agree	A. Strongly agree	B. No	A. Yes	A. Yes	B. No
41	18 - 24	Male	TRUE	TRUE	TRUE	TRUE	E. Strongly disagree	B. Agree	E. Strongly disagree	B. Agree	E. Strongly disagree	A. Strongly agree	A. Strongly agree	B. No	A. Yes	A. Yes	A. Yes
42	18 - 24	Male	TRUE	TRUE	FALSE	TRUE	A. Strongly agree	B. Agree	D. Disagree	A. Strongly agree	C. Neutral	A. Strongly agree	A. Strongly agree	B. No	A. Yes	A. Yes	A. Yes
43	18 - 24	Female	TRUE	TRUE	FALSE	TRUE	B. Agree	B. Agree	D. Disagree	A. Strongly agree	B. Agree	A. Strongly agree	A. Strongly agree	B. No	A. Yes	A. Yes	B. No
44	18 - 24	Female	TRUE	TRUE	TRUE	TRUE	B. Agree	B. Agree	B. Agree	B. Agree	E. Strongly disagree	B. Agree	A. Strongly agree	B. No	A. Yes	A. Yes	B. No
45	18 - 24	Female	TRUE	TRUE	TRUE	TRUE	B. Agree	C. Neutral	B. Agree	A. Strongly agree	E. Strongly disagree	A. Strongly agree	A. Strongly agree	B. No	A. Yes	A. Yes	B. No
46	18 - 24	Male	TRUE	TRUE	TRUE	TRUE	B. Agree	B. Agree	B. Agree	B. Agree	E. Strongly disagree	A. Strongly agree	A. Strongly agree	B. No	C. Sometimes	C. Sometimes	A. Yes
47	18 - 24	Female	TRUE	TRUE	TRUE	TRUE	B. Agree	B. Agree	B. Agree	D. Disagree	E. Strongly disagree	A. Strongly agree	A. Strongly agree	B. No	C. Sometimes	C. Sometimes	A. Yes
48	18 - 24	Male	TRUE	TRUE	TRUE	TRUE	A. Strongly agree	C. Neutral	B. Agree	B. Agree	D. Disagree	A. Strongly agree	A. Strongly agree	B. No	A. Yes	A. Yes	B. No
49	18 - 24	Male	TRUE	TRUE	TRUE	TRUE	B. Agree	B. Agree	B. Agree	A. Strongly agree	E. Strongly disagree	B. Agree	A. Strongly agree	B. No	C. Sometimes	C. Sometimes	C. Sometimes
50	18 - 24	Female	TRUE	TRUE	TRUE	TRUE	A. Strongly agree	B. Agree	D. Disagree	A. Strongly agree	E. Strongly disagree	B. Agree	A. Strongly agree	B. No	C. Sometimes	A. Yes	B. No
51	25 - 34	Female	TRUE	TRUE	TRUE	TRUE	B. Agree	A. Strongly agree	B. Agree	A. Strongly agree	D. Disagree	A. Strongly agree	A. Strongly agree	B. No	C. Sometimes	C. Sometimes	A. Yes
52	18 - 24	Female	TRUE	TRUE	TRUE	TRUE	D. Disagree	B. Agree	B. Agree	A. Strongly agree	E. Strongly disagree	A. Strongly agree	A. Strongly agree	B. No	A. Yes	C. Sometimes	C. Sometimes
53	18 - 24	Female	TRUE	TRUE	FALSE	TRUE	B. Agree	E. Strongly disagree	E. Strongly disagree	A. Strongly agree	D. Disagree	A. Strongly agree	A. Strongly agree	B. No	A. Yes	A. Yes	B. No
54	18 - 24	Female	TRUE	TRUE	TRUE	May be	B. Agree	C. Neutral	D. Disagree	D. Disagree	C. Neutral	A. Strongly agree	A. Strongly agree	B. No	C. Sometimes	A. Yes	A. Yes
55	18 - 24	Male	TRUE	TRUE	TRUE	TRUE	B. Agree	B. Agree	E. Strongly disagree	B. Agree	E. Strongly disagree	A. Strongly agree	B. Agree	B. No	C. Sometimes	A. Yes	B. No
56	18 - 24	Female	TRUE	TRUE	TRUE	TRUE	B. Agree	A. Strongly agree	A. Strongly agree	A. Strongly agree	E. Strongly disagree	A. Strongly agree	A. Strongly agree	C. Sometimes	A. Yes	C. Sometimes	A. Yes
57	18 - 24	Male	TRUE	TRUE	May be	TRUE	C. Neutral	B. Agree	C. Neutral	A. Strongly agree	D. Disagree	A. Strongly agree	A. Strongly agree	B. No	C. Sometimes	C. Sometimes	A. Yes
58	18 - 24	Male	TRUE	TRUE	May be	TRUE	B. Agree	A. Strongly agree	E. Strongly disagree	B. Agree	E. Strongly disagree	A. Strongly agree	B. Agree	B. No	A. Yes	A. Yes	A. Yes
59	18 - 24	Male	TRUE	FALSE	TRUE	TRUE	A. Strongly agree	B. Agree	A. Strongly agree	C. Neutral	A. Strongly agree	A. Strongly agree	A. Strongly agree	C. Sometimes	A. Yes	A. Yes	B. No
60	18 - 24	Male	TRUE	TRUE	TRUE	TRUE	A. Strongly agree	B. Agree	A. Strongly agree	B. Agree	D. Disagree	A. Strongly agree	B. Agree	C. Sometimes	A. Yes	C. Sometimes	B. No
61	18 - 24	Male	TRUE	TRUE	FALSE	TRUE	A. Strongly agree	B. Agree	B. Agree	A. Strongly agree	E. Strongly disagree	A. Strongly agree	A. Strongly agree	A. Yes	B. No	A. Yes	A. Yes
62	18 - 24	Female	TRUE	TRUE	TRUE	TRUE	B. Agree	B. Agree	C. Neutral	A. Strongly agree	A. Strongly agree	A. Strongly agree	E. Strongly disagree	B. No	A. Yes	A. Yes	B. No
63	18 - 24	Female	TRUE	FALSE	TRUE	TRUE	A. Strongly agree	A. Strongly agree	B. Agree	B. Agree	C. Neutral	A. Strongly agree	C. Neutral	C. Sometimes	A. Yes	A. Yes	B. No
64	18 - 24	Male	TRUE	TRUE	TRUE	TRUE	C. Neutral	B. Agree	A. Strongly agree	C. Neutral	E. Strongly disagree	B. Agree	B. Agree	C. Sometimes	A. Yes	A. Yes	B. No
65	18 - 24	Female	TRUE	TRUE	TRUE	TRUE	C. Neutral	A. Strongly agree	B. Agree	C. Neutral	E. Strongly disagree	A. Strongly agree	C. Neutral	C. Sometimes	A. Yes	A. Yes	B. No
66	18 - 24	Male	TRUE	TRUE	TRUE	TRUE	B. Agree	A. Strongly agree	A. Strongly agree	E. Strongly disagree	E. Strongly disagree	B. Agree	B. Agree	C. Sometimes	A. Yes	C. Sometimes	C. Sometimes
67	18 - 24	Female	TRUE	TRUE	TRUE	TRUE	B. Agree	C. Neutral	A. Strongly agree	B. Agree	E. Strongly disagree	B. Agree	B. Agree	C. Sometimes	A. Yes	A. Yes	C. Sometimes
68	18 - 24	Male	TRUE	TRUE	TRUE	TRUE	B. Agree	B. Agree	B. Agree	A. Strongly agree	D. Disagree	A. Strongly agree	B. Agree	C. Sometimes	C. Sometimes	B. No	B. No
69	18 - 24	Male	TRUE	TRUE	FALSE	TRUE	B. Agree	B. Agree	B. Agree	A. Strongly agree	D. Disagree	B. Agree	C. Neutral	B. No	C. Sometimes	A. Yes	C. Sometimes
70	18 - 24	Female	TRUE	TRUE	May be	TRUE	B. Agree	B. Agree	C. Neutral	B. Agree	D. Disagree	B. Agree	A. Strongly agree	B. No	C. Sometimes	C. Sometimes	C. Sometimes
71	18 - 24	Male	TRUE	TRUE	FALSE	TRUE	B. Agree	C. Neutral	C. Neutral	A. Strongly agree	E. Strongly disagree	B. Agree	C. Neutral	C. Sometimes	A. Yes	B. No	C. Sometimes
72	18 - 24	Male	TRUE	FALSE	TRUE	May be	B. Agree	C. Neutral	A. Strongly agree	C. Neutral	E. Strongly disagree	C. Neutral	C. Neutral	C. Sometimes	A. Yes	C. Sometimes	C. Sometimes
73	18 - 24	Male	TRUE	TRUE	May be	May be	B. Agree	C. Neutral	C. Neutral	C. Neutral	D. Disagree	B. Agree	A. Strongly agree	A. Yes	A. Yes	B. No	C. Sometimes
74	18 - 24	Female	TRUE	TRUE	FALSE	TRUE	B. Agree	D. Disagree	D. Disagree	B. Agree	D. Disagree	B. Agree	B. Agree	B. No	A. Yes	A. Yes	B. No
75	18 - 24	Male	TRUE	FALSE	TRUE	TRUE	B. Agree	B. Agree	B. Agree	D. Disagree	C. Neutral	B. Agree	A. Strongly agree	B. No	A. Yes	A. Yes	A. Yes
76	18 - 24	Female	TRUE	TRUE	TRUE	TRUE	B. Agree	D. Disagree	D. Disagree	B. Agree	D. Disagree	B. Agree	A. Strongly agree	B. No	C. Sometimes	A. Yes	A. Yes
77	18 - 24	Female	TRUE	TRUE	TRUE	TRUE	C. Neutral	C. Neutral	D. Disagree	D. Disagree	E. Strongly disagree	A. Strongly agree	A. Strongly agree	C. Sometimes	A. Yes	A. Yes	A. Yes
78	18 - 24	Male	TRUE	TRUE	May be	TRUE	C. Neutral	B. Agree	D. Disagree	B. Agree	D. Disagree	A. Strongly agree	A. Strongly agree	A. Yes	B. No	A. Yes	A. Yes
79	18 - 24	Male	TRUE	TRUE	May be	TRUE	B. Agree	C. Neutral	D. Disagree	B. Agree	E. Strongly disagree	B. Agree	A. Strongly agree	B. No	A. Yes	A. Yes	B. No
80	18 - 24	Male	TRUE	TRUE	FALSE	TRUE	D. Disagree	E. Strongly disagree	E. Strongly disagree	B. Agree	B. Agree	B. Agree	A. Strongly agree	B. No	A. Yes	A. Yes	A. Yes
81	18 - 24	Male	TRUE	TRUE	FALSE	TRUE	B. Agree	D. Disagree	E. Strongly disagree	A. Strongly agree	D. Disagree	A. Strongly agree	A. Strongly agree	C. Sometimes	C. Sometimes	A. Yes	B. No
82	18 - 24	Male	TRUE	TRUE	TRUE	TRUE	A. Strongly agree	C. Neutral	B. Agree	B. Agree	C. Neutral	B. Agree	B. Agree	B. No	A. Yes	A. Yes	C. Sometimes
83	18 - 24	Male	TRUE	TRUE	TRUE	TRUE	B. Agree	C. Neutral	B. Agree	C. Neutral	C. Neutral	B. Agree	B. Agree	B. No	A. Yes	A. Yes	A. Yes
84	18 - 24	Female	TRUE	TRUE	TRUE	TRUE	D. Disagree	B. Agree	E. Strongly disagree	C. Neutral	D. Disagree	A. Strongly agree	C. Neutral	B. No	C. Sometimes	C. Sometimes	A. Yes
85	18 - 24	Male	TRUE	TRUE	FALSE	TRUE	B. Agree	C. Neutral	B. Agree	A. Strongly agree	D. Disagree	C. Neutral	B. Agree	B. No	B. No	A. Yes	A. Yes
86	18 - 24	Male	TRUE	TRUE	May be	TRUE	B. Agree	B. Agree	D. Disagree	C. Neutral	D. Disagree	A. Strongly agree	B. Agree	B. No	A. Yes	A. Yes	A. Yes
87	25 - 34	Male	TRUE	TRUE	TRUE	TRUE	B. Agree	D. Disagree	D. Disagree	B. Agree	D. Disagree	B. Agree	B. Agree	B. No	A. Yes	A. Yes	A. Yes
88	18 - 24	Female	TRUE	TRUE	TRUE	TRUE	A. Strongly agree	E. Strongly disagree	D. Disagree	A. Strongly agree	D. Disagree	A. Strongly agree	A. Strongly agree	B. No	A. Yes	A. Yes	B. No
89	18 - 24	Male	TRUE	TRUE	FALSE	TRUE	B. Agree	B. Agree	D. Disagree	D. Disagree	D. Disagree	A. Strongly agree	A. Strongly agree	B. No	A. Yes	A. Yes	B. No
90	25 - 34	Male	May be	TRUE	TRUE	TRUE	C. Neutral	B. Agree	D. Disagree	B. Agree	B. Agree	B. Agree	A. Strongly agree	B. No	A. Yes	A. Yes	A. Yes
91	18 - 24	Female	TRUE	TRUE	FALSE	TRUE	A. Strongly agree	A. Strongly agree	A. Strongly agree	A. Strongly agree	E. Strongly disagree	A. Strongly agree	A. Strongly agree	B. No	A. Yes	A. Yes	C. Sometimes
92	18 - 24	Male	TRUE	TRUE	FALSE	TRUE	D. Disagree	B. Agree	D. Disagree	B. Agree	E. Strongly disagree	A. Strongly agree	A. Strongly agree	B. No	A. Yes	A. Yes	A. Yes
93	18 - 24	Male	TRUE	TRUE	TRUE	TRUE	B. Agree	D. Disagree	C. Neutral	B. Agree	C. Neutral	A. Strongly agree	B. Agree	B. No	A. Yes	A. Yes	B. No
94	18 - 24	Male	TRUE	TRUE	TRUE	TRUE	D. Disagree	A. Strongly agree	B. Agree	A. Strongly agree	C. Neutral	B. Agree	C. Neutral	B. No	A. Yes	A. Yes	A. Yes
95	18 - 24	Female	TRUE	May be	FALSE	May be	D. Disagree	C. Neutral	C. Neutral	B. Agree	C. Neutral	B. Agree	A. Strongly agree	B. No	B. No	A. Yes	A. Yes
96	18 - 24	Male	TRUE	TRUE	FALSE	TRUE	C. Neutral	D. Disagree	E. Strongly disagree	A. Strongly agree	C. Neutral	A. Strongly agree	A. Strongly agree	B. No	A. Yes	A. Yes	B. No
97	18 - 24	Female	TRUE	TRUE	TRUE	TRUE	B. Agree	C. Neutral	C. Neutral	A. Strongly agree	D. Disagree	A. Strongly agree	A. Strongly agree	B. No	A. Yes	A. Yes	B. No
98	25 - 34	Female	TRUE	TRUE	TRUE	TRUE	C. Neutral	C. Neutral	C. Neutral	B. Agree	D. Disagree	B. Agree	B. Agree	B. No	A. Yes	A. Yes	B. No
99	18 - 24	Male	TRUE	TRUE	TRUE	TRUE	E. Strongly disagree	C. Neutral	D. Disagree	C. Neutral	E. Strongly disagree	A. Strongly agree	A. Strongly agree	B. No	A. Yes	A. Yes	B. No
100	18 - 24	Female	TRUE	TRUE	TRUE	TRUE	A. Strongly agree	C. Neutral	E. Strongly disagree	A. Strongly agree	E. Strongly disagree	A. Strongly agree	A. Strongly agree	B. No	A. Yes	A. Yes	B. No
101	18 - 24	Male	TRUE	TRUE	TRUE	TRUE	C. Neutral	A. Strongly agree	C. Neutral	E. Strongly disagree	B. Agree	B. Agree	B. Agree	B. No	A. Yes	A. Yes	A. Yes
102	18 - 24	Female	TRUE	TRUE	TRUE	TRUE	C. Neutral	D. Disagree	C. Neutral	A. Strongly agree	D. Disagree	A. Strongly agree	A. Strongly agree	B. No	C. Sometimes	A. Yes	B. No
103	18 - 24	Female	TRUE	TRUE	FALSE	May be	B. Agree	D. Disagree	D. Disagree	B. Agree	D. Disagree	C. Neutral	B. Agree	B. No	A. Yes	A. Yes	B. No
104	18 - 24	Male	TRUE	TRUE	FALSE	TRUE	A. Strongly agree	E. Strongly disagree	D. Disagree	A. Strongly agree	D. Disagree	B. Agree	D. Disagree	B. No	B. No	A. Yes	C. Sometimes
105	18 - 24	Male	TRUE	TRUE	TRUE	TRUE	D. Disagree	C. Neutral	D. Disagree	A. Strongly agree	D. Disagree	A. Strongly agree	A. Strongly agree	B. No	C. Sometimes	A. Yes	B. No
106	25 - 34	Male	TRUE	TRUE	TRUE	TRUE	A. Strongly agree	E. Strongly disagree	E. Strongly disagree	A. Strongly agree	E. Strongly disagree	A. Strongly agree	A. Strongly agree	B. No	A. Yes	A. Yes	B. No
107	18 - 24	Male	TRUE	May be	FALSE	TRUE	B. Agree	A. Strongly agree	D. Disagree	B. Agree	D. Disagree	C. Neutral	B. Agree	B. No	A. Yes	A. Yes	A. Yes
108	18 - 24	Male	TRUE	TRUE	May be	TRUE	D. Disagree	B. Agree	C. Neutral	B. Agree	D. Disagree	B. Agree	A. Strongly agree	C. Sometimes	B. No	A. Yes	A. Yes
109	18 - 24	Male	TRUE	TRUE	May be	TRUE	A. Strongly agree	C. Neutral	B. Agree	A. Strongly agree	B. Agree	A. Strongly agree	A. Strongly agree	B. No	C. Sometimes	C. Sometimes	A. Yes
110	18 - 24	Male	TRUE	TRUE	May be	TRUE	D. Disagree	A. Strongly agree	A. Strongly agree	A. Strongly agree	B. Agree	A. Strongly agree	A. Strongly agree	B. No	B. No	C. Sometimes	A. Yes
111	18 - 24	Female	TRUE	TRUE	TRUE	TRUE	B. Agree	B. Agree	D. Disagree	A. Strongly agree	A. Strongly agree	A. Strongly agree	A. Strongly agree	C. Sometimes	C. Sometimes	C. Sometimes	A. Yes
112	18 - 24	Female	TRUE	TRUE	FALSE	TRUE	A. Strongly agree	C. Neutral	D. Disagree	B. Agree	E. Strongly disagree	B. Agree	A. Strongly agree	B. No	C. Sometimes	C. Sometimes	C. Sometimes
113	18 - 24	Male	TRUE	TRUE	FALSE	TRUE	D. Disagree	B. Agree	E. Strongly disagree	A. Strongly agree	E. Strongly disagree	A. Strongly agree	A. Strongly agree	C. Sometimes	C. Sometimes	B. No	A. Yes
114	18 - 24	Male	TRUE	TRUE	FALSE	TRUE	B. Agree	A. Strongly agree	E. Strongly disagree	A. Strongly agree	C. Neutral	A. Strongly agree	A. Strongly agree	B. No	B. No	C. Sometimes	A. Yes
115	18 - 24	Male	TRUE	TRUE	TRUE	TRUE	D. Disagree	B. Agree	C. Neutral	A. Strongly agree	B. Agree	A. Strongly agree	B. Agree	B. No	A. Yes	B. No	B. No
116	18 - 24	Male	TRUE	TRUE	FALSE	May be	A. Strongly agree	C. Neutral	B. Agree	A. Strongly agree	B. Agree	A. Strongly agree	A. Strongly agree	B. No	A. Yes	B. No	A. Yes
117	18 - 24	Male	TRUE	TRUE	TRUE	TRUE	A. Strongly agree	B. Agree	A. Strongly agree	B. Agree	D. Disagree	A. Strongly agree	A. Strongly agree	C. Sometimes	C. Sometimes	C. Sometimes	A. Yes
118	18 - 24	Male	May be	TRUE	May be	TRUE	B. Agree	B. Agree	A. Strongly agree	E. Strongly disagree	A. Strongly agree	A. Strongly agree	A. Strongly agree	B. No	A. Yes	C. Sometimes	C. Sometimes
119	18 - 24	Female	TRUE	May be	TRUE	May be	A. Strongly agree	A. Strongly agree	A. Strongly agree	A. Strongly agree	C. Neutral	A. Strongly agree	A. Strongly agree	C. Sometimes	B. No	B. No	C. Sometimes
120	18 - 24	Female	TRUE	TRUE	FALSE	TRUE	A. Strongly agree	B. Agree	D. Disagree	A. Strongly agree	A. Strongly agree	A. Strongly agree	B. Agree	A. Yes	A. Yes	C. Sometimes	A. Yes
121	18 - 24	Male	TRUE	TRUE	FALSE	TRUE	B. Agree	B. Agree	C. Neutral	A. Strongly agree	C. Neutral	A. Strongly agree	A. Strongly agree	B. No	A. Yes	A. Yes	A. Yes
122	25 - 34	Male	TRUE	TRUE	TRUE	TRUE	A. Strongly agree	D. Disagree	A. Strongly agree	A. Strongly agree	E. Strongly disagree	A. Strongly agree	A. Strongly agree	C. Sometimes	A. Yes	A. Yes	B. No
123	18 - 24	Male	May be	TRUE	TRUE	TRUE	C. Neutral	C. Neutral	A. Strongly agree	B. Agree	D. Disagree	B. Agree	B. Agree	B. No	A. Yes	A. Yes	A. Yes

## References

[1] (2024). World Health Organization (WHO). Antimicrobial Resistance. https://www.who.int/news-room/fact-sheets/detail/antimicrobial-resistance.

[2] (2024). World Health Organization (WHO). Global Antimicrobial Resistance and Use Surveillance System (‎GLASS)‎ Report. https://www.who.int/publications/i/item/9789240062702.

[3] (2024). National Centre for Disease Control (NCDC). National Action Plan on AMR (NAP-AMR). https://ncdc.mohfw.gov.in/national-action-plan-on-amr-nap-amr/.

[4] (2024). Centers for Disease Control and Prevention. Antimicrobial Resistance. https://www.cdc.gov/ncezid/dfwed/keyprograms/antimicrobial-resistance.html.

[5] Murray CJ, Ikuta KS, Sharara F (2022). Global burden of bacterial antimicrobial resistance in 2019: a systematic analysis. Lancet.

[6] Akalu SD, Belavadi NG (2017). Awareness of antibiotic usage and antimicrobial resistance among interns in a tertiary care hospital. Int J Basic Clin Pharmacol.

[7] Kulkarni P, Kuruvilla A, Roy R (2017). An evaluation of knowledge, attitude and practice of rational antibiotic usage and antibiotic resistance among interns in a teaching tertiary care hospital: A cross-sectional questionnaire based study. Indian J Pharm Pharmacol.

[8] Khan A K A, Banu G, K K R (2013). Antibiotic resistance and usage-a survey on the knowledge, attitude, perceptions and practices among the medical students of a southern Indian teaching hospital. J Clin Diagn Res.

[9] Ganesh M, Sridevi SA, Paul CM (2014). Antibiotic use among medical and para medical students: knowledge, attitude and its practice in a tertiary health care centre in Chennai - a scientific insight. Medical Science.

[10] Huang Y, Gu J, Zhang M (2013). Knowledge, attitude and practice of antibiotics: a questionnaire study among 2500 Chinese students. BMC Med Educ.

[11] Lv B, Zhou Z, Xu G (2014). Knowledge, attitudes and practices concerning self-medication with antibiotics among university students in western China. Trop Med Int Health.

[12] Sakeena MH, Bennett AA, Jamshed S, Mohamed F, Herath DR, Gawarammana I, McLachlan AJ (2018). Investigating knowledge regarding antibiotics and antimicrobial resistance among pharmacy students in Sri Lankan universities. BMC Infect Dis.

[13] Albalawi L, Alhawiti AS, Alnasser D (2023). Knowledge, attitudes, and practices among pharmacy and non-pharmacy interns in Saudi Arabia regarding antibiotic use and antibiotic resistance: a cross-sectional descriptive study. Healthcare (Basel).

[14] Jha N, Shrestha S, Shankar PR (2020). Knowledge, attitude and practice about antibiotic use, self-medication and antibiotic resistance among final year medical students and interns at a medical college in Lalitpur, Nepal. J Chitwan Med College.

[15] Kanyike AM, Olum R, Kajjimu J (2022). Antimicrobial resistance and rational use of medicine: knowledge, perceptions, and training of clinical health professions students in Uganda. Antimicrob Resist Infect Control.

[16] Nisabwe L, Brice H, Umuhire MC (2020). Knowledge and attitudes towards antibiotic use and resistance among undergraduate healthcare students at University of Rwanda. J Pharm Policy Pract.

[17] Asharani N, Dhanalakshmi TA, Shyamanth M (2020). Knowledge, attitude, and practices toward antibiotic usage and antibiotic resistance among medical students and interns: a cross-sectional study. J Med Sci Heal.

[18] (2023). OpenEpi: Open-Source Epidemiologic Statistics for Public Health, Version 3. http://www.OpenEpi.com.

